# Progress in Obstetrics

**Published:** 1902-03-08

**Authors:** 


### Progress in Obstetrics.

**Movements of the Pelvic Bones during the Operation of Symphyseotomy.**—A. C. Sandstein[1] arrived at the following conclusions from a study of symphyseotomy on 28 recent female cadavera. The first movement was that of the pubes outwards, by rotation of the innominate bones on vertical axes passing through their respective sacro-iliac joints. The second movement consisted in rotation of the innominate bones on a horizontal transverse axis passing through the sacrum, and carried the pubes downwards. The third movement consisted in the rotation of the innominate bone on its own long axis, so as to cause the ilium to become more erect. An examination of all the results showed that the diameters lying more or less transversely were increased greatly by each centimetre of pubic separation. But unfortunately it is more usually the true conjugate that presents the difficulty, and he found that 6 centimetres of pubic separation enlarged that only about 1 centimetre. As a result of his investigations, Dr. Sandstein stated that pubic separation should be limited to 6 centimetres, and as each centimetre increased the true conjugate by 1·67 milimetres, the utmost amount of increase of the true conjugate which could be aimed at was 1 centimetre, or two-fifths of an inch.

**Rupture of the Uterus.**—R. Milne Murray [2] reports a very interesting and instructive case of spontaneous rupture of an apparently normal uterus at the commencement of labour. Nine hours after the rupture occurred the abdomen was opened, notwithstanding that the patient was in a profound state of collapse. On making the incision an enormous quantity of blood and liquor amnii escaped. The uterus was firmly retracted, its contents entirely in the abdominal cavity, and a tear found extending from a point about an inch behind the left fallopian tube downwards and forwards along the border of the uterus to the vaginal roof. Near the lower end of the rent the line of laceration turned sharply towards the front, and involved the left upper region of the bladder. It was decided that removal of the uterus would give the patient the best chance. The rent in the bladder was repaired by several fine catgut stitches. The operation occupied rather more than an hour, and at this stage the patient's condition was so serious that it was decided to dispense with the abdominal toilet; the abdomen therefore was closed with silkworm gut, although the peritoneum contained much blood, both fluid and coagulated, a considerable amount of liquor amnii, and a very obvious amount of meconium and vernix caseosa. Milne Murray draws attention to the fact that the peritoneum is capable of dealing with a large amount of blood and other materials provided that they are not septic. No explanation could be given as to why this uterus ruptured, yet there must have been some obscure change in the quality of the uterine muscle. Durlacher [3] reports a case of rupture of the uterus, in which, after attempting to deliver the patient with forceps, version was performed, and as the head would not follow, such forcible traction was used that it tore off and remained in the uterus. On examination Durlacher found the stump of the neck, the chin, and the face of the fœtus inside the uterus, while the vault of the head protruded through a rupture of that organ. An attempt was made to bring down the head with a blunt hook introduced into the mouth, but as this was unsuccessful laparotomy was performed, the patient died however a few minutes after the completion of the operation. Durlacher considered that seeing the conjugate vera only measured $2\frac{1}{3}$ inches, craniotomy should have been performed instead of attempting to deliver by forceps or by version. He thinks that although the condition of the patient when he was called in was so critical that laparotomy was little likely to be attended with success, yet it gave the patient the only chance of recovery.

1 Lancet, Jan. 18, 1902. 2 Brit. Med. Journ., Jan. 11, 1902. 3 Brit. Med. Jour., Feb. 1, 1902.

